# Neurofibromatosis-Noonan Syndrome With Primary Amenorrhoea: A Case Report

**DOI:** 10.7759/cureus.42098

**Published:** 2023-07-18

**Authors:** Ipsita Mohapatra, Subha R Samantaray

**Affiliations:** 1 Obstetrics and Gynecology, All India Institute of Medical Sciences, Kalyani, Kalyani, IND; 2 Obstetrics and Gynecology, Prathima Institute of Medical Science, Karimnagar, IND

**Keywords:** café-au-lait spots, primary amenorrhea, rasopathy, noonan, neurofibromatosis

## Abstract

Neurofibromatosis-Noonan syndrome is a rare RASopathy syndrome. It occurs due to the mutation in the NF1 gene and the patients present with the phenotypic features of both Neurofibromatosis and Noonan syndrome. Here a case of an early adolescent girl is described who presented with the chief complaint of primary amenorrhoea and on evaluation was diagnosed to be a patient of Neurofibromatosis-Noonan syndrome. The index case was short-statured with a short and broad neck. Physical examination revealed a pointed pinna, hypertelorism, telecanthus, characteristic facies, and multiple freckles all over the body. She also had numerous atypical café-au-lait spots. Whole genome sequencing revealed Neurofibromatosis-Noonan syndrome which was likely a pathogenic variant causative of the typical phenotype present with a mutation in the neurofibromin gene (NF1) on chromosome 17q11. We discuss here the management and follow-up of the case.

## Introduction

Neurofibromatosis-Noonan syndrome (NFNS) is a rare RASopathy syndrome. It was first described by Allanson et al. in 1985 [1]. NFNS occurs due to the mutation in the NF1 gene and the patients present with the phenotypic features of both Neurofibromatosis (NF) and Noonan syndrome (NS).

NF is an autosomal dominant disorder that presents with variable mutations. It has an incidence of about 1:2500 to 1:3000 [2] and is diagnosed based on the criteria laid down by the National Health Institute. NF patients typically present with the characteristic café-au-lait spots and neurofibromas.

NS is also an autosomal dominant disorder and it has varied phenotypical manifestations which keep on changing with age. The most consistent features are wide-set eyes, low-set ears, short stature, and pulmonic stenosis. Gene mutations of the RAAS/MAPK (mitogen-activated protein kinase) signaling pathway are involved [3]. Its incidence is about 1 in 1000 to 2500 [4].

We present here a case of an early adolescent girl who presented with the chief complaint of primary amenorrhoea and on evaluation was diagnosed to be a patient of NFNS.

## Case presentation

**Figure 1:**
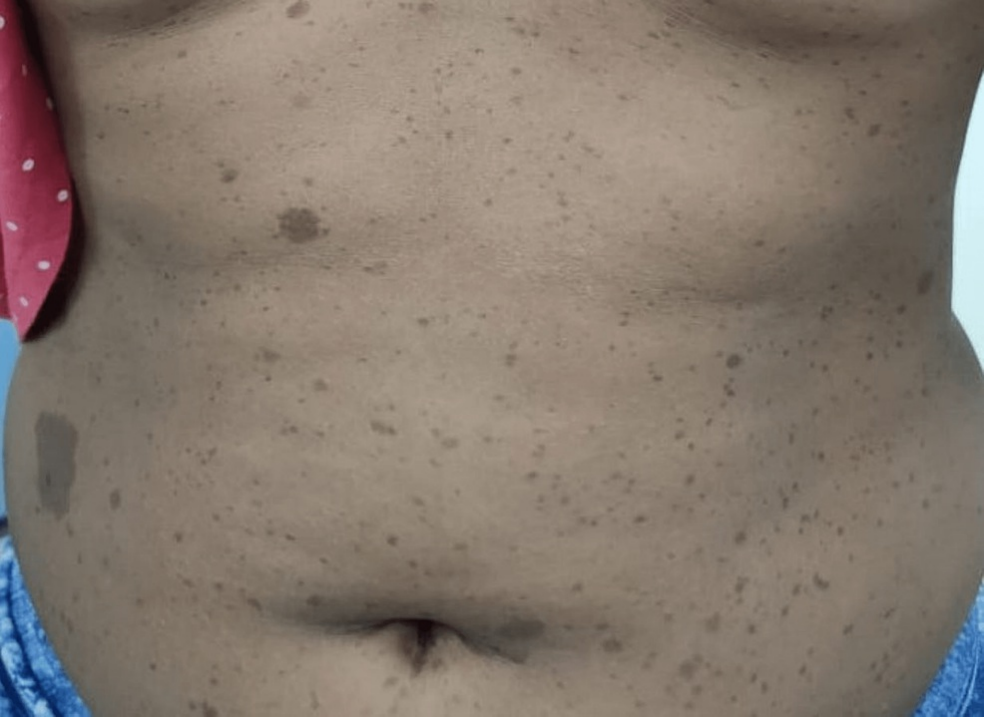
Atypical café-au-lait (<15mm) spots

An early adolescent girl at the age of 16 years presented to the gynecology outpatient clinic with the chief complaint of primary amenorrhoea. She was short-statured (total height 145.9cm) with a short and broad neck. Her weight was 54 kilograms. She had pointed pinna, hypertelorism, telecanthus, and characteristic facies. On examination of her external body, she had multiple axillary, inguinal, and palmar freckles. Numerous asymptomatic café-au-lait spots were distributed over the whole body, predominantly over the trunk. Most of these were atypical café-au-lait spots (irregular, smudgy borders) with sizes <15mm (Figure 1). These were absent at birth and had developed over the last three to four years.

There was no evidence of neurofibromas and no intellectual disability was noted. She didn’t have any other systemic manifestations, no bony abnormalities, or any developmental delay. The index case had no history of any bleeding diathesis and no complaints of vision. On physical examination, no subcutaneous or plexiform neurofibromata were found, and upon slit lamp examination, no Lisch’s nodules could be detected. On electrocardiogram, no cardiac abnormality was noted.

There was also a history of third and fourth ventriculomegaly about six months back with no associated blurring of vision, headache, seizures, or focal neural deficit. Magnetic resonance imaging done at that time revealed moderate communicating hydrocephalus with periventricular interstitial edema. Conservative management was done and the patient was discharged in four days. At the present time, examination revealed no neurological deficits.

On examination for the development of secondary sexual characters, there were no axillary and pubic hairs present. Breast development was Tanner stage 4, with Tanner sexual maturity scoring B4A0P0 (breast development 4, axillary hairs 0, pubic hairs 0). The external genitalia was normally developed and on gentle per speculum examination cervix was found to be present.

Investigations for hormonal profiling were ordered which revealed low levels of serum follicle-stimulating hormone (0.05mIU/mL), serum luteinizing hormone (<0.5mIU/mL), and serum estrogen (<10pg/mL). Karyotyping revealed a total of 44 autosomes and two sex chromosomes with the presence of a large heterochromatin region on one of chromosome 16(46XX,16qh+) which was reported as a normal heterotrophic variation. Due to strong clinical suspicion, whole genome sequencing was done (Table 1). It revealed NFNS which was likely a pathogenic variant causative of the typical phenotype present with a mutation in the neurofibromin gene (NF1) on chromosome 17q11. Ultrasonography revealed a normal uterus and ovaries.

**Table 1:** Whole genome sequencing report

Gene & Transcript	Variant	Location	Zygosity	Disorder (OMIM)	Inheritance	Classification
NF1 NM_000267.3	c.5592_5596delTTTAA (p.Asn1864Lysfs*26)	Exon 38	Heterozygous	Neurofibromatosis-Noonan syndrome NFNS (601321)	Autosomal Dominant	Likely Pathogenic

Due to the presence of hypogonadotropic hypogonadism, the patient was started on cyclical estradiol valerate (2mg) daily for 21 days and norethisterone acetate (10mg) in the second half of the cycle for 10 days every month. The patient started having regular cycles of hormonal therapy. The patient is on regular follow-ups with a multidisciplinary team consisting of a gynecologist, dermatologist, ophthalmologist, and neurosurgeon. During therapy with estrogen and progesterone, she was closely monitored for any new appearance of neurofibromata or any problem of visual acuity.

Written informed consent was obtained from the patient and her parents for the publication of this case report and images of the patient and her genetic tests.

## Discussion

NS and NF are two distinct syndromes in the RASopathy spectrum. NF type I (NF1) is caused by a heterozygous mutation in the neurofibromin gene (NF1) on chromosome 17q11 that codes for a protein called neurofibromin which is a tumor suppressor gene. In NS, the genes that undergo mutation are involved in the cell signaling pathway of RAS/MAPK. The most common mutation found in NS is the mutation at the PTPN11 gene [5]. Both these conditions are inherited in an autosomal dominant manner, thus, parents who have any of these syndromes have a possibility of passing the mutation on to the children in 50% of cases. NS can also occur due to de novo mutation [5].

The diagnosis of NF1 can be made using the NIH diagnostic criteria. According to this, the patient must meet at least two criteria among the following criteria: ≥6 café-au-lait spots (>5 mm diameter in pre-pubertal individuals; >15 mm in postpubertal individuals); ≥2 neurofibromas of any type, or 1 plexiform neurofibroma; optic glioma; ≥2 Lisch nodules or hamartomas; a distinct osseous lesion (sphenoid dysplasia; tibial pseudoarthrosis); axillary or inguinal regions frecklings; ≥1 first degree relative who has type 1 NF [6].

The diagnostic criteria for NS include major and minor measures [7]. The major criteria include facial dysmorphism, pulmonic valve stenosis, cardiomyopathy, height less than the third percentile, pectus excavatum/carinatum, first-degree relative diagnosed with the NS, and intellectual disability. In our patient, there was no history of any family member suffering from a similar disease. Minor criteria include height less than the 10th percentile, broad thorax, mild facial dysmorphism, and first-degree relative with suggestive NS.

One of the suspicious features of NS is increased nuchal translucency in the intrauterine period. During childhood, there may be developmental delay, strabismus, and hearing defects [8]. As the child grows, typical facies may develop, along with chest dysmorphology, heart problems like hypertrophic cardiomyopathy, and intellectual disability. Dysplastic lymphatic changes can occur leading to early generalized lymphoedema in the abdomen and the extremities. Some patients may present with bleeding diatheses. The index case presented with typical facies, but had no other deformities or disabilities.

Among the NFNS patients, mutations in both PTPN11 and N1 have been found in many. However, some patients may present with no detectable PTPN11 mutation. In our patient also, only a mutation in the neurofibromin gene (NF1) on chromosome 17q11 was found. NFNS presents with additional phenotypical characteristics of NS, such as short stature, short broad neck, low-set ears, and ocular hypertelorism [5].

The index case was treated with estrogen and progesterone therapy to address the correction of primary amenorrhea, to which she responded. However, this therapy is postulated to increase the size of subcutaneous and plexiform neurofibromas [9]. Due to the activation of microglia by an estrogen-mediated pathway and a gender-specific role for cAMP regulation in glioma genesis, there is a possibility of the development of optic glioma resulting in loss of vision. The dose of progesterone has been limited to a short period of 10 days every month to reduce the risk of tumor development. Due to these concerns, regular follow-up of the patient with the dermatologist, neurologist, and ophthalmologist is being done at six-monthly intervals.

## Conclusions

Patients presenting with primary amenorrhea and phenotypic features of both NF and NS should be considered for the possibility of NFNS. These cases need detailed investigations to rule out cardiac abnormalities and neurological problems. If diagnosed at an early stage, these cases may be considered for growth hormone therapy as the patients are usually short-statured. These patients should be put under regular follow-up with the cardiologist, dermatologist, and gynecologist for close surveillance and to detect any complications due to prolonged hormone therapy.

## References

[1] Allanson JE, Hall JG, Van Allen MI (1985). Noonan phenotype associated with neurofibromatosis. Am J Med Genet.

[2] Ferner RE, Huson SM, Thomas N (2007). Guidelines for the diagnosis and management of individuals with neurofibromatosis 1. J Med Genet.

[3] Rauen KA (2013). The RASopathies. Annu Rev Genomics Hum Genet.

[4] Myers A, Bernstein JA, Brennan ML (2014). Perinatal features of the RASopathies: Noonan syndrome, cardiofaciocutaneous syndrome and Costello syndrome. Am J Med Genet A.

[5] Jett K, Friedman JM (2010). Clinical and genetic aspects of neurofibromatosis 1. Genet Med.

[6] Legius E, Messiaen L, Wolkenstein P (2021). Revised diagnostic criteria for neurofibromatosis type 1 and Legius syndrome: an international consensus recommendation. Genet Med.

[7] Bhambhani V, Muenke M (2014). Noonan syndrome. Am Fam Physician.

[8] van der Burgt I (2007). Noonan syndrome. Orphanet J Rare Dis.

[9] Dugoff L, Sujansky E (1996). Neurofibromatosis type 1 and pregnancy. Am J Med Genet.

